# Leaf Chlorophyll Content Estimation of Winter Wheat Based on Visible and Near-Infrared Sensors

**DOI:** 10.3390/s16040437

**Published:** 2016-03-25

**Authors:** Jianfeng Zhang, Wenting Han, Lvwen Huang, Zhiyong Zhang, Yimian Ma, Yamin Hu

**Affiliations:** 1 College of Information Engineering, Northwest A&F University, Yangling 712100, China; springchilly@gmail.com (L.H.); zzy@nwsuaf.edu.cn (Z.Z.); 15339142186@163.com (Y.M.); yaminexc@gmail.com (Y.H.); 2 Institute of Water Saving Agriculture in Arid Area of China, Northwest A&F University, Yangling 712100, China

**Keywords:** winter wheat, leaf chlorophyll content, visible and near infrared sensors, agricultural information acquisition, partial least squares

## Abstract

The leaf chlorophyll content is one of the most important factors for the growth of winter wheat. Visual and near-infrared sensors are a quick and non-destructive testing technology for the estimation of crop leaf chlorophyll content. In this paper, a new approach is developed for leaf chlorophyll content estimation of winter wheat based on visible and near-infrared sensors. First, the sliding window smoothing (SWS) was integrated with the multiplicative scatter correction (MSC) or the standard normal variable transformation (SNV) to preprocess the reflectance spectra images of wheat leaves. Then, a model for the relationship between the leaf relative chlorophyll content and the reflectance spectra was developed using the partial least squares (PLS) and the back propagation neural network. A total of 300 samples from areas surrounding Yangling, China, were used for the experimental studies. The samples of visible and near-infrared spectroscopy at the wavelength of 450,900 nm were preprocessed using SWS, MSC and SNV. The experimental results indicate that the preprocessing using SWS and SNV and then modeling using PLS can achieve the most accurate estimation, with the correlation coefficient at 0.8492 and the root mean square error at 1.7216. Thus, the proposed approach can be widely used for winter wheat chlorophyll content analysis.

## 1. Introduction

Winter wheat is one of the most important crops in the north of China, and it is usually cultivated with the right amount of nitrogen to achieve a high output. Therefore, nitrogen content is an important indicator of the level of plant nutrition for winter wheat [1]. Studies showed that plant chlorophyll content was positively correlated with nitrogen content [2]. Thus, the value of leaf chlorophyll content can help to understand nutritional status of the plant, and scientifically guide the fertilization management to ensure a good crop quality and yield [3,4]. This practice has an important significance for the modern precision agriculture.

Generally, there are two methods to measure the leaf chlorophyll concentration: destructive testing and nondestructive testing (NDT) [5]. Spectrophotometric method, a traditional destructive method used in the laboratory, is based on the technique that measures leaf chlorophyll concentration by organic extraction and spectrophotometric analysis. This destructive approach is accurate and is considered as a benchmark for the estimation of chlorophyll content. However, it requires special equipment, which is expensive and time consuming [6]. Therefore, it could not meet the needs of rapid and non-destructive testing. Spectroscopy technique based on the visible and near-infrared spectroscopy could be applied to estimate the chlorophyll content as a rapid and non-destructive method [7], Ulissi *et al.* [8] proved that the chlorophyll spectra range of the 496–694 nm was highly correlated with the analyzed leaf N concentration, and reported a portable spectrophotometer of N concentration of tomato leaves based on visible and near-infrared spectroscopy (VIS-NIR). The use of spectrometry sensors for crop nutrition measure has been extensively studied [8,9,10,11]. Holer *et al.* [12] studied the relationship between the value of the spectrum and the chlorophyll concentrations, and proposed the role of red edge position for the vegetation chlorophyll concentration estimation. Fang *et al.* [13] used samples of rape leaves, proposed a model to predict the relative leaf chlorophyll content using two parameters of red edge position and the peak position of the green spectral band, and demonstrated that it was feasible to predict the relative leaf chlorophyll content by spectral analysis.

However, various practical operational factors, as well as physical and natural properties (for example, the surface scattering and optical path change because of the size of the solid particle on the wheat leaf), affect the reflection spectra, and thus obscure the extraction of the quantitative information. By using mathematical correction methods or preprocessing methods, a considerable amount of such unwanted changes may be removed from the spectral data [14]. Both the standard normal variable transformation (SNV) and the multiplicative scatter correction (MSC) are often used in the simultaneous correction of additive and multiplicative effects on spectra [15]. Preprocessing is very important to obtain the robust and accurate quantitative information for spectroscopy sensors.

Partial least squares (PLS) and BP neural network (BPNN) algorithm have been used in spectral data analysis modeling for prediction in various systems. PLS is a linear and multivariate analysis method widely used in spectral data analysis [7], while BPNN is a powerful method to solve the nonlinear problems of classification and regression analysis [16,17]. Jamshidi *et al.* [18] used MSC and SNV to process spectral data, and applied the PLS modeling method to the non-destructive estimation model for Valencia oranges taste characteristics based on the visible and near-infrared spectroscopy. Chu *et al.* [19] used visible and near-infrared sensor image to estimate the soluble protein content of oilseed rape leaves, where genetic algorithm–partial least square (GAPLS) was used for sensitive wavelength selection. Yao *et al.* [20] studied the relationship between rice chlorophyll content and spectral data, compared the effects of PLS, SMLR, PCR and BPNN modeling methods, and proved that the rice leaf pigment PLS model of near infrared spectroscopy could achieve better performance. Because PLS algorithm and BPNN algorithm have contributed to the modeling application for spectral analysis of agricultural products, this paper takes these two algorithms to study the relationship between the relative chlorophyll content and the spectral data of winter wheat leaf, and modeling analysis.

Studies on the relative chlorophyll content of non-destructive testing mainly focus on chlorophyll value (Soil and Plant Analysis Development, SPAD) measurement, which estimates the crop relative chlorophyll content by averaging all the values of one point SPAD measured repeatedly. SPAD values express the relative amounts of chlorophyll in crop leaves and have been demonstrated in several studies [21,22,23].

In this paper, samples of winter wheat leaves were chosen from three different regions in Shaanxi Province, China, the relationship between the relative chlorophyll content and the spectral data of crop leaf in a selected area is studied. Then, the quantitative analysis model is developed and its efficiency is verified. The rest of this paper is organized as follows, after representing the collection of the large number of samples used for studies, this paper addresses the preprocessing methods of the spectral data. The next section describes the quantitative analysis model for the relation between the leaf relative chlorophyll content and the reflectance spectra. This paper then presents the experimental results and analysis. The final section discusses the research conclusions, and presents the proposed approach for winter wheat leaf chlorophyll content analysis.

## 2. Materials and Methods

**Figure 1:**
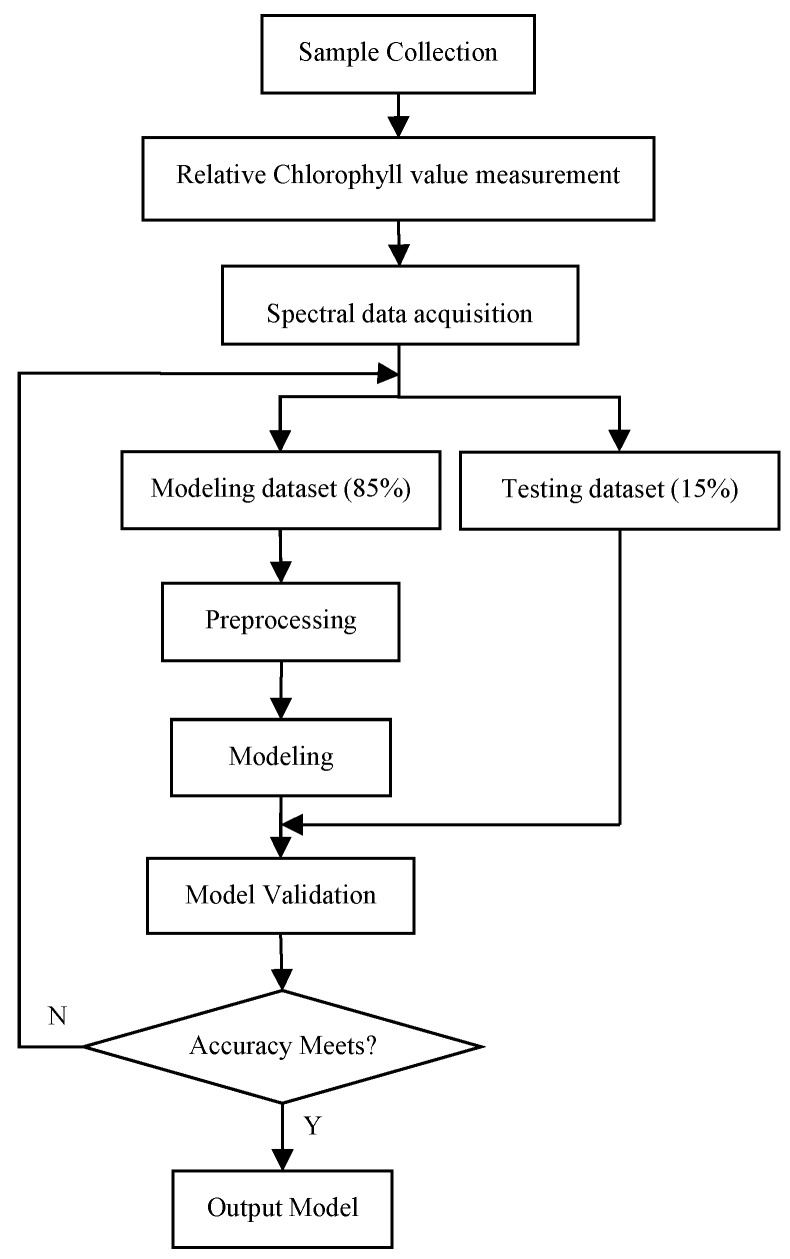
Flowchart of modeling and analyzing for leaf chlorophyll content estimation of winter wheat based on visible and near-infrared spectroscopy.

The experimental materials used in this paper are winter wheat leaves selected from three different regions in Shaanxi, China. The flowchart of modeling and analyzing for leaf chlorophyll content estimation of winter wheat based on visible and near-infrared spectroscopy is given in Figure 1, which gives a new approach to the study of wheat spectrum NDT.

### 2.1. Sample Collections

The experimental winter wheat leaves are selected from three different areas surrounding Yangling town of Shaanxi Province in China. The three areas are the Arid and Semi-arid Agriculture Institute of China (ASAIC), Juliang Farm with 200 hectares of grain base (JLFarm) and Rougu Town with about 135 hectares of grain base (RGTown). A number of 100 sample leaves in each region, totally 300 samples, was chosen. The diversity of samples, which covers different areas of arid and semi-arid and various influencing factors for the growth of wheat, can well avoid the problem of single condition farmland and single sample modeling. Samples were collected from 15 to 30 March 2014, which is the wheat jointing duration. A certain area region of every sample was selected, circled and taken the field measurement of the chlorophyll content. Then, each leaf was put into a sample storage bag marked with a unique number. Finally, the fresh samples picked with standard correct agricultural sample collection methods were taken back to the Spectroscopy Laboratory in College of Information Engineering in Northwest A&F University, China for scanning hyperspectral images. The blades can remain fresh within 24 h.

### 2.2. Data Acquisition

In the experimental fields, the chlorophyll value of a certain area size of 1.43 cm^2^ was measured by CM-1000 at the distance of 30.5 cm and marked the measurement position. The CM-1000, used to measure the value of wheat chlorophyll in the fields, is a handheld chlorophyll meter produced by the Spectrum Technologies, Inc., Aurora, IL, USA. At the distance of 30.5–183.0 cm, CM-1000 measures the relative chlorophyll content of a certain area of the blade by the perception of 700 nm and 840 nm reflection light. The size of the relative chlorophyll content is from 0 to 999 SPAD.

Then, the spectral data of the fresh samples was collected immediately using ImSpector N10E high-spectrometer (SPECIM-Hyperspectral Imaging Solutions Company with Global Presence, Oulu, Finland). Firstly, the lens was adjusted to focus on the object samples at the distance of 30.5 cm, the translation stage and spin platform were set up, and the scan mode was chosen. Secondly, the black and white were focused and corrected combining with the software. Finally, the leaves were placed on the stage and scanned to obtain the hyperspectral image of the blades.

**Figure 2:**
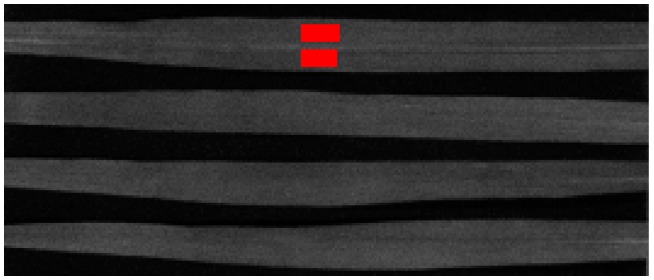
Hyperspectral image of a juxtaposition of four winter wheat leaves and an interest region marked in red.

**Figure 3:**
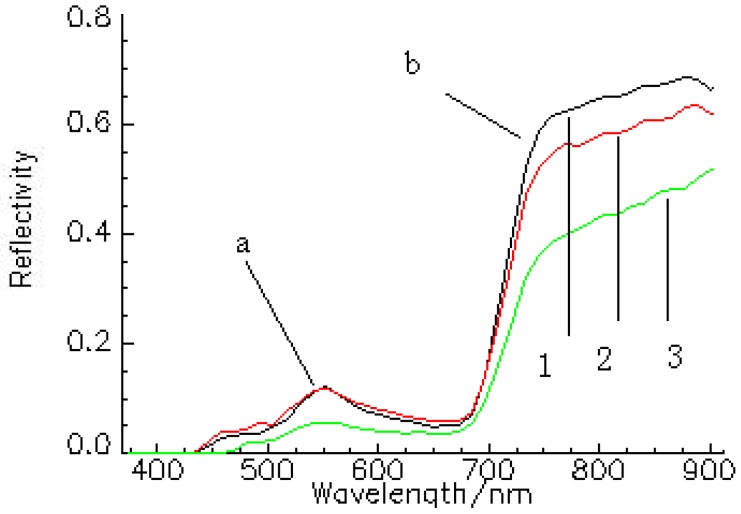
Raw reflectance spectra of samples. The first reflectance spectrum is one sample of Juliang Farm, the second is one sample of Rougu Town, and the third is one sample of the Arid and Semi-arid Agriculture Institute of China. The peak at about 550 nm (**a**) represents the green light reflection region. The band at 690–720 nm; (**b**) represents the near-infrared to red edge region.

An example of the marked part selected from the hyperspectral image is given in Figure 2, which shows a juxtaposition of four winter wheat leaves. For chlorophyll content measurement, a rectangular area was selected and divided into two parts to avoid the veins, and the average spectral reflectance image of the region was obtained [24]. In this research work, a range of 450–900 nm wavelength reflectance spectral data was selected for data analysis and model. The raw reflectance spectra of samples are shown in Figure 3, where the abscissa is the spectral wavelength and the vertical axis is the spectral reflection coefficient. The reflection peak of about 550 nm is the green light reflection region. The bandlength of 690–720 nm is the red edge region, which shows a negative correlation between its peak sizes and the chlorophyll content [22].

### 2.3. Preprocessing of Reflectance Spectra

In the spectral analysis, it is an important step to use an appropriate method to carry on the data preprocess. The main purpose of the preprocessing to the winter wheat leaf spectrum is to eliminate the influence of the prediction models, and various types of preprocessing are used to compare different preprocessing methods for obtaining knowledge of the performance and the suitability of different preprocessing methods when applied to the reflection spectra.

**Figure 4:**
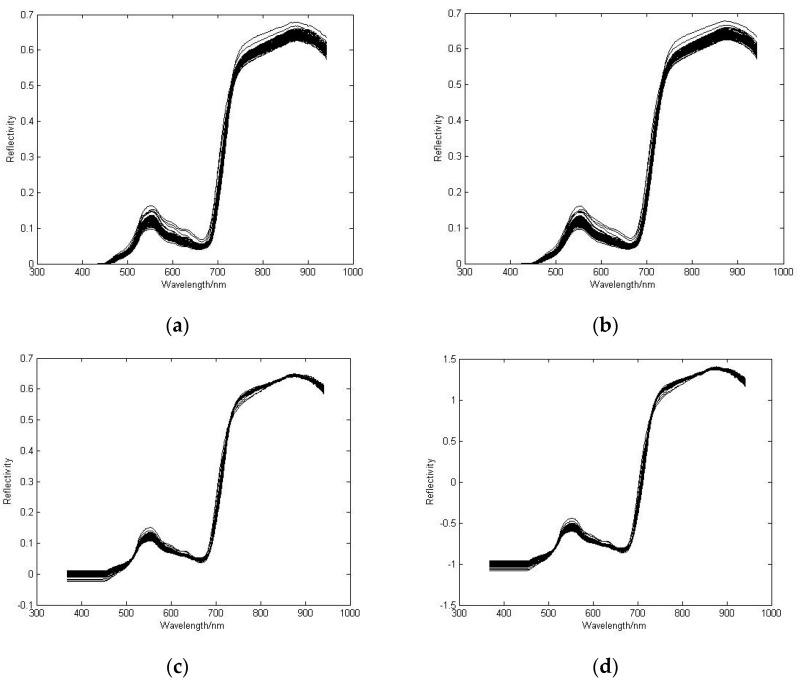
Raw reflectance spectrum (**a**) preprocessed by the sliding window smoothing; (**b**), the sliding window smoothing and multiplicative scatter correction; (**c**), and the sliding window smoothing and standard normal variable transformation; (**d**) The samples were from the Arid and Semi-arid Agriculture Institute, Yangling, China.

Some preprocessing methods more commonly used for spectrum are Smoothing, SNV, MSC, and other derivatives [15,18]. SWS (Sliding Window Smoothing) is a weighted average method to reduce noise of the spectral images, thereby improving the signal to noise ratio. MSC has a good effect in solving the problem of non-uniform particle size on the surface of the samples. SNV is an effective solution for measuring the change of light [18]. In this research, the spectral images were preprocessed by SWS, MSC, SNV, SWS in combination with MSC (SWS-MSC) and SWS in combination with SNV (SWS-SNV). Figure 4a–d shows the raw reflectance spectrum (**a**) preprocessed by the sliding window smoothing; (**b**), the sliding window smoothing and multiplicative scatter correction; (**c**), the sliding window smoothing and standard normal variable transformation; (**d**), where the samples were from the Arid and Semi-arid Agriculture Institute, Yangling, China. It can be seen that the absorbance difference between samples is significantly reduced. This difference can be approximately considered as limitation only by the content of the difference caused by the material composition and are the results of interactions with the near-infrared absorption of all components of the samples. The influence of particle size has been eliminated and the scattering effect has been correspondingly reduced. To quantify the preprocessing effect, the reflectance spectra were applied to predict the chlorophyll content using PLS model. The best preprocessing method was chosen by the prediction.

### 2.4. Prediction Model Using PLS

PLS regression is a principal component regression statistical method. It is a mathematical optimization technology to find a linear model to represent the forecasting variables and observed variables into a new space. Today, PLS regression is most widely used in the field of the spectral data analysis. In order to obtain the best modeling effect, PLS simultaneously analyze the spectral matrix and the concentration of the matrix decomposition, and the relationship between them is also considered.

**Figure 5:**
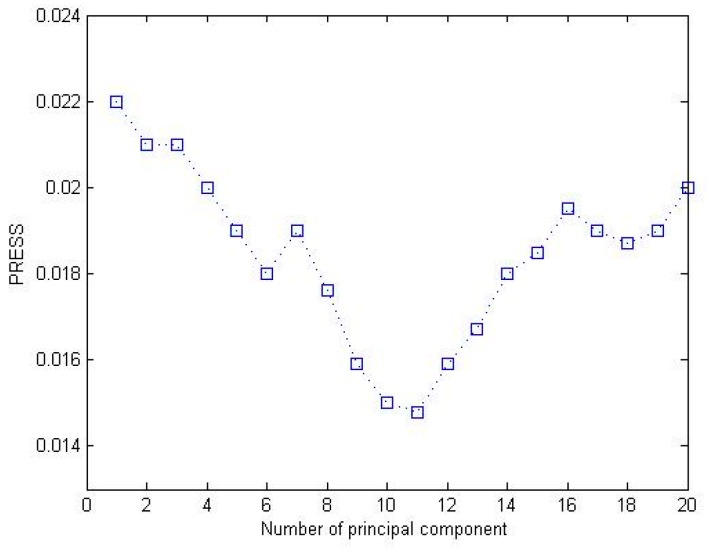
Effects of different principal components.

After the spectral data were preprocessed, the 300 samples were classified into three groups according to the region sources. For the 100 samples in each group, 85 samples and 15 samples were randomly chosen as the calibration sets and the prediction sets, respectively. The quantitative analysis model between chlorophyll values and spectral data was established in the band length range of 450–900 nm based on PLS, and the SPAD values of the prediction dataset then were predicted. The number of the principal components was selected by interactive testing. The prediction residual error sum of square (P_RESS_) was used as the evaluation criteria [23]. P_RESS_ was modeled by a certain number of the principal components, the samples were predicted, and the differences between the predicted values and the measured values were calculated. P_RESS_ is defined as

(1) $$P_{RESS}=\sum_{i=1}^{n}\sum_{j=1}^{d}{\left(p_{ij}-r_{ij}\right)}^{2}$$

where *n* is the number of the calibration dataset samples; *d* is the number of the principal components for the model; $p_{ij}$ is the sample predictive value; and $r_{ij}$ is the measured value of the sample. The model has better predictive ability with smaller P_RESS_ value. Figure 5 shows the relationship between PRESS and the different principal components of ASAIC samples, where the horizontal axis is the principal components and the vertical axis is PRESS value. It can be seen that the number of principal components is 11 when PRESS reaches the minimum value. Using the principal component number of 11, the cumulative contribution rate of PLS analysis is 96.52%.

### 2.5. Prediction Model by BPNN

Neural network is a statistical learning mechanism neurologically inspired. It has a strong pattern recognition capability, which enables it to learn to represent a complex system with multivariable inputs and outputs. BPNN is a popular neural network, which has the advantages of nonlinearity, parallel processing, fault-tolerance, self-adaptation, and self-learning. Therefore, the BPNN is the incomparably superior in a variety of applications including prediction, data fitting, classification, and system modeling.

A typical BPNN has an input layer, one or more hidden layer(s), and an output layer. The input layer is a layer which is connected with the external environment, and the condition of training the neural network should be represented. The output layer is actually a model for the external environment, and the number of the output neurons is directly related to the type of the task. The hidden layer is a group of neurons that have an activation function, and provide an intermediate layer between the input and the output layer. BPNN algorithm is designed to minimize the root mean square error of a multi-layered feed forward perception of the actual output and the desired output.

In the hidden layer, the number of node has a great influence on the performance of the BPNN. If the number of neurons are less to the complexity of the work, it cannot fully reflect the relationship between the input and output variables. If more unnecessary neurons are to be set in the network, over-fitting may occur. Usually, the number of neurons in the hidden layer is determined by the empirical Equation (2), and the influence of different neuron numbers on the prediction of the model:

(2) $$n_{1}=\sqrt{n+m}+a$$

where $n_{1}$ is the number of neurons in the hidden layer; *n* is the number of input neurons; *m* is the number of output neurons; and *a* is a constant between 1 to 10.

**Figure 6:**
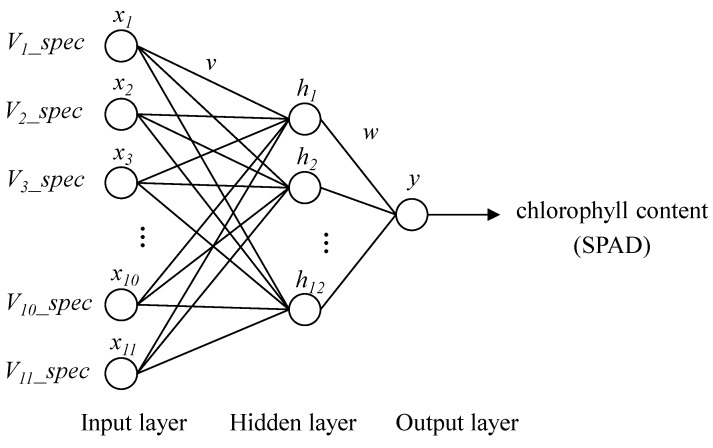
Structure of the proposed neural network for prediction of the chlorophyll content of wheat leaf.

The proposed neural network shown in Figure 6 which has one hidden layer, is to predict using 11 spectral variables (*V_i__spec*, *i* = 1, … 11,) as the input vector and measuring the chlorophyll content of wheat leaf as the output variable. The 11 spectral variables as the input vector were the reflectivity of the wavelength at 501.2 nm, 535.0 nm, 550.5 nm, 575.0 nm, 711.4 nm, 728.2 nm, 749.8 nm, 769.0 nm, 788.2 nm, 841.0 nm and 886.6 nm. The intermediate hidden layer, which has the number of 12 nodes, uses the *tansig* as the activation function, and the output layer uses the *purelin* function.

## 3. Experiments

In this study, 300 spectral images of winter wheat leaves total were selected to develop the model of the leaf chlorophyll content. The prediction performance was evaluated by the root mean squared error (R_MSE_) and correlation coefficient (*R*^2^).

### 3.1. PLS

**Figure 7:**
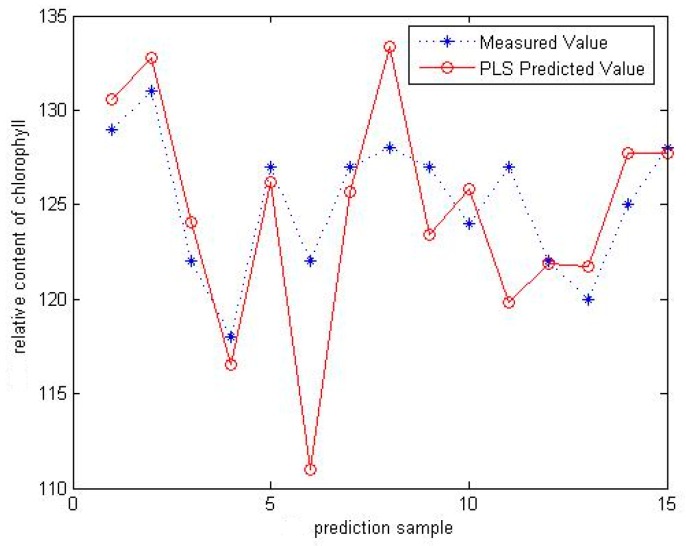
Measurement and prediction values of testing dataset using PLS.

After the sample spectral data were processed by SWS-MSC, the predictive model using PLS was applied to predict the chlorophyll content of each 15 testing samples according to the different region source respectively. The measured and predicted values of 15 testing samples of ASAIC samples are shown in Figure 7, where the horizontal axis is the number of the samples and the vertical axis is the relative content of chlorophyll, in which the *R*^2^ of the model is 0.8429 and the R_MSE_ is 1.7369.

### 3.2. BPNN

**Figure 8:**
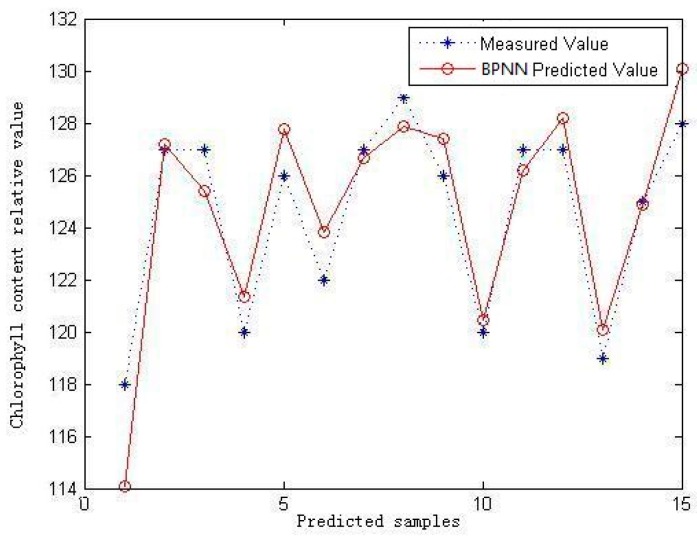
Measurement and prediction values of testing dataset using BPNN.

The BPNN model is trained using the *trainlm* function. The training requirement accuracy is 0.001, the maximum number of iterations is 1000, and the learning rate is 0.01. The predicted results for the samples using the BPNN predicted model are shown in Figure 8, in which the *R*^2^ is 0.8482 and the R_MSE_ is 1.7940.

### 3.3. Results

The accuracy of the predicted value was evaluated by the correlation coefficient *R*^2^, and the root mean square error R_MSE_ [25]. R_MSE_ is calculated by:

(3) $$R_{MSE}=\sqrt{\frac{\sum_{i=1}^{n}{\left(y_{i}-{\hat{y}}_{i}\right)}^{2}}{n}}$$

where $y_{i}$ is the measured value of sample *i*; ${\hat{y}}_{i}$ is the predicted value; and *n* is the number of samples.

Table 1, Table 2 and Table 3 illustrate the predictive capabilities of the model for three group sample datasets, respectively. It can be seen that the predictive performances achieved by using SWS-MSC and SWS-SNV are better than those achieved by using MSC and SNV individually. The best result, the *R*^2^ is larger than 0.8492 and the R_MSE_ is smaller than 1.7216, can be reached by using SWS-SNV. Experimental results indicate that spectroscopy analysis can be applied to predict the chlorophyll content of a certain area crop leaf and that both the model and methods have the advantage of universality.

## 4. Conclusions

Comparing the predictive efficiency based on PLS and BPNN model, and the preprocessing using MSC, SNV, SWS-MSC, and SWS-SNV, it can be seen that the preprocessing using SWS-MSC or SWS-SNV is better than only using MSC or SNV regardless whether the different parts of the sample datasets or the model. The experimental and comparison results indicate that the combination of multiple preprocessing for spectral data is an effective way to improve the accuracy, and the preprocessing using SWS-SNV and then modeling using PLS can achieve the most accurate estimation with the correlation coefficient at 0.8492 and the root mean square error at 1.7216. The predictive model performances are good, and it can well meet the actual demand of the crop leaf relative chlorophyll content NDT instead of averaging a certain number of single point values. The experimental results show that the proposed approach using the PLS model with SWS-SNV preprocessing is feasible to predict the relative chlorophyll content of a certain area of winter wheat leaf leaf area based on the visible and near-infrared spectroscopy sensors, and can achieve a better precision and accuracy. For the further study, the proposed method could be applied to an available hand-held chlorophyll instrument or an on-line field application.

## Figures and Tables

**Table 1:** Predictive capabilities of testing dataset from the Arid and Semi-arid Agriculture Institute of China (ASAIC) preprocessing by different method and modeling using the Partial least squares (PLS) or BP neural network (BPNN): correlation coefficient (R^2^), and root mean square error (R_MSE_).

Preprocessing Method	Model	R^2^	R_MSE_
MSC	BPNN	0.8256	1.9785
SNV	BPNN	0.8141	1.8945
MSC	PLS	0.8127	1.7269
SNV	PLS	0.8116	1.7996
SWS-MSC	BPNN	0.8482	1.7940
SWS-SNV	BPNN	0.8454	1.7970
SWS-MSC	PLS	0.8429	1.7369
SWS-SNV	PLS	0.8492	1.7216

**Table 2:** Predictive capabilities of testing dataset from Juliang Farm.

Preprocessing Method	Model	R^2^	R_MSE_
MSC	BPNN	0.9287	1.4984
SNV	BPNN	0.9294	1.6215
MSC	PLS	0.9258	1.5487
SNV	PLS	0.9125	1.4956
SWS-MSC	BPNN	0.9548	1.5959
SWS-SNV	BPNN	0.9521	1.7532
SWS-MSC	PLS	0.9518	1.3535
**SWS-SNV**	**PLS**	**0.9597**	**1.3281**

**Table 3:** Predictive capabilities of testing dataset from Rougu Town.

Preprocessing Method	Model	R^2^	R_MSE_
MSC	BPNN	0.9027	1.9210
SNV	BPNN	0.8994	1.8561
MSC	PLS	0.9026	1.6894
SNV	PLS	0.9158	1.5962
SWS-MSC	BPNN	0.9171	1.7760
SWS-SNV	BPNN	0.9137	1.7184
SWS-MSC	PLS	0.9269	1.5972
**SWS-SNV**	**PLS**	**0.9279**	**1.5948**
